# Women in radiology: gender diversity is not a metric—it is a tool for excellence

**DOI:** 10.1007/s00330-019-06493-1

**Published:** 2019-12-04

**Authors:** Rahel A. Kubik-Huch, Valérie Vilgrain, Gabriel P. Krestin, Maximilian F. Reiser, Ulrike I. Attenberger, Ada U. Muellner, Christopher P. Hess, Hedvig Hricak

**Affiliations:** 1 Department of Medical Services, Institute of Radiology, Kantonsspital Baden, CH-5404 Baden, Switzerland; 2 APHP, HUPNVS, Hôpital Beaujon, 100 bd General Leclerc, 92110 Clichy, France; 3 Université de Paris, Paris, France; 4 Department of Radiology and Nuclear Medicine at Erasmus MC, University Medical Center Rotterdam, Room Ne-515k, P.O. Box 2040, 3000 CA Rotterdam, The Netherlands; 5 Department of Radiology, Ludwig-Maximilians-University, Marchioninistr. 15, 81377 Munich, Germany; 6 Institute of Clinical Radiology and Nuclear Medicine, University Medical Center Mannheim, Theodor-Kutzer-Ufer 1-3, 68167 Mannheim, Germany; 7 Department of Radiology, Memorial Sloan Kettering Cancer Center, 1275 York Avenue, c-278, New York, NY 10065 USA; 8 Department of Radiology and Biomedical Imaging, University of California, San Francisco, 505 Parnassus Avenue, Room M-392, UCSF, Box 0628, San Francisco, CA 94143-0628 USA

**Keywords:** Leadership, Female, Workforce, Radiology, Mentoring

## Abstract

**Abstract:**

*Women in Focus: Be Inspired* was a unique programme held at the 2019 European Congress of Radiology that was structured to address a range of topics related to gender and healthcare, including leadership, mentoring and the generational progression of women in medicine. In most countries, women constitute substantially fewer than half of radiologists in academia or private practice despite frequently accounting for at least half of medical school enrolees. Furthermore, the proportion of women decreases at higher academic ranks and levels of leadership, a phenomenon which has been referred to as a “leaky pipeline”. Gender diversity in the radiologic workplace, including in academic and leadership positions, is important for the present and future success of the field. It is a tool for excellence that helps to optimize patient care and research; moreover, it is essential to overcome the current shortage of radiologists. This article reviews the current state of gender diversity in academic and leadership positions in radiology internationally and explores a wide range of potential reasons for gender disparities, including the lack of role models and mentorship, unconscious bias and generational changes in attitudes about the desirability of leadership positions. Strategies for both individuals and institutions to proactively increase the representation of women in academic and leadership positions are suggested.

**Key Points:**

*• Gender-diverse teams perform better. Thus, gender diversity throughout the radiologic workplace, including in leadership positions, is important for the current and future success of the field.*

*• Though women now make up roughly half of medical students, they remain underrepresented among radiology trainees, faculty and leaders.*

*• Factors leading to the gender gap in academia and leadership positions in Radiology include a lack of role models and mentors, unconscious biases, other societal barriers and generational changes.*

## Introduction

**Fig. 1:**
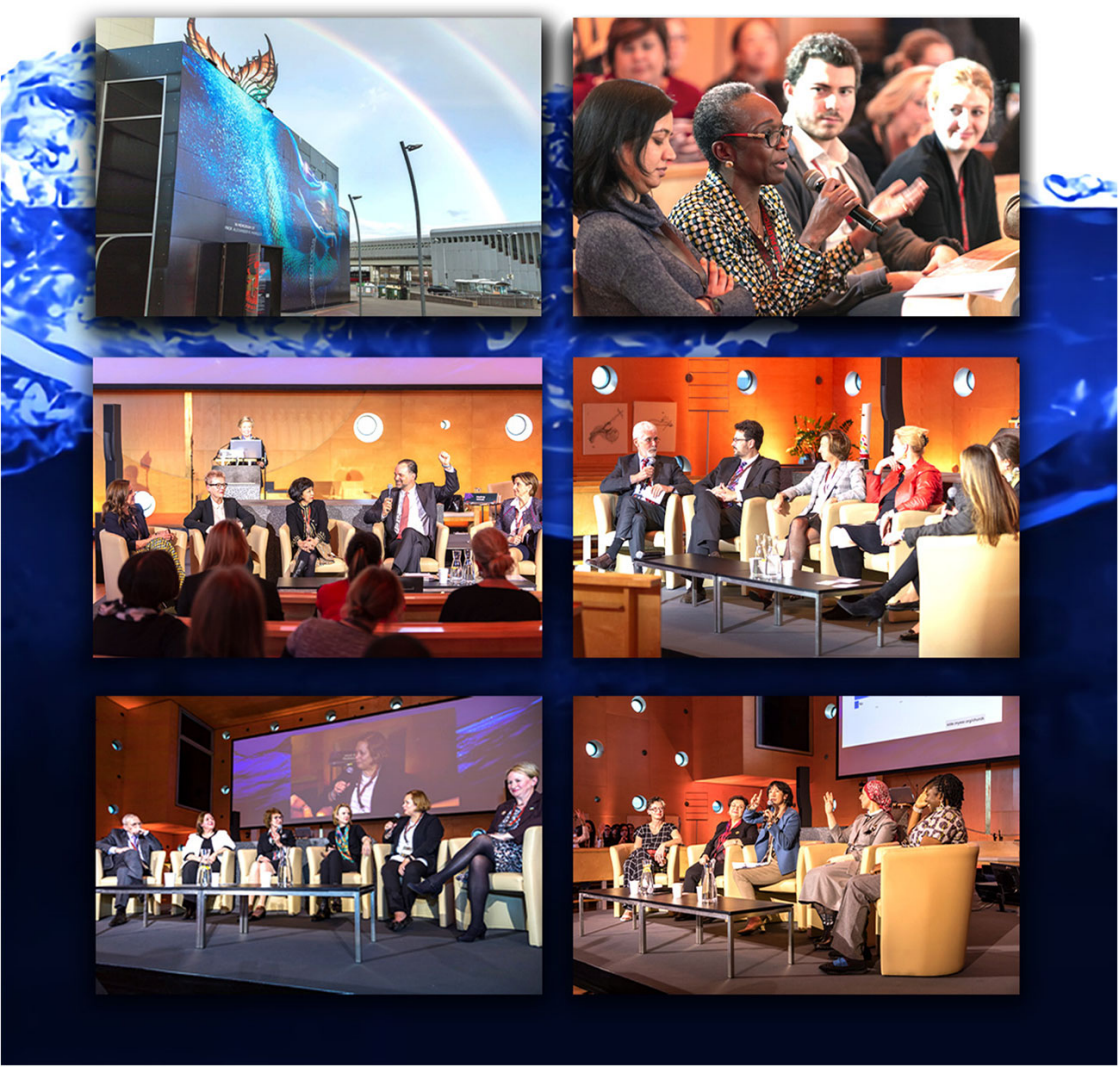
Impressions from the programme “Women in Focus: Be Inspired” during the 2019 European Congress of Radiology in Vienna

*Women in Focus: Be Inspired* was a new programme featured at the 2019 European Congress of Radiology in Vienna (Fig. 1). The event was dedicated to the memory of Professor Alexander R. Margulis, a role model and mentor who was ahead of his time in supporting and promoting female leadership [1]. Prominent female and male professionals from academic medicine, public health and the healthcare industry shared their views on topics relating to female leadership. Drawing on ideas discussed during the programme, this article surveys the situation of women in Radiology internationally, focusing on their careers in academia and leadership and highlighting the value of gender diversity for optimizing the quality of clinical care and research. Persistent gaps in gender diversity, and their potential causes, are reviewed, and strategies to enhance the representation of women in academic and leadership positions in Radiology are proposed.

## Diversity in the workforce: a tool for excellence

The hypothesis of this article is that increasing the representation of women in academic and leadership positions in Radiology strengthens the specialty as a whole.

Research in multiple industries has illustrated that including people with diverse genders, skill sets and viewpoints in decision-making improves workforce engagement, critical analytical thinking and innovation [2–4]. In the corporate world, it is increasingly recognized that including women in leadership results in greater competitiveness and economic success [4, 5]. Women now constitute approximately half or more of medical students in the USA and Europe [6–8]. As such, increasing their representation in academic and leadership positions in Radiology is crucial to ensuring that the field draws on the largest and richest possible talent pool, offers the highest quality of care and produces innovations that address the needs of male and female patients equally.

## Radiology: the current representation of women in academia and in leadership positions

To put the proportions of women radiologists in academia and leadership positions in perspective, it helps to consider the distribution of women in the broader field of Radiology. A recent survey of 29 professional radiological organizations in 26 countries indicated that, based on the organizations’ membership compositions, approximately 33.5% of radiologists were women [6]. This proportion varied widely between countries and was lowest in the USA, at 27.2%. In Romania, Spain and Thailand, women made up more than 50% of radiologists, and in a majority of countries, they accounted for at least one third.

The membership of the European Society of Radiology (ESR) provides another illustrative example of women’s representation in Radiology. Currently, in 2019, 30% of ESR members are women. Within this contingent, there are substantial generational differences. While only 13% of “traditionalists” (born 1927–1945) are female, the percentage rises with each successive generation, reaching 41% among generation Y members (born 1977–1992) (based on research undertaken by the ESR, 2019, Table 1).

**Table 1:** Overview of the composition of the European Society of Radiology (ESR) membership in 2019, stratified by gender and generation (based on research undertaken by the European Society of Radiology, 2019)

	Traditionalists	Baby boomers	Generation X	Generation Y (millenials)	All generations
	Born 1927–1945	Born 1946 to 1964	Born 1965 to 1976	Born 1977 to 1992	
	Aged 74 to 92	Aged 55 to 73	Aged 43 to 54	Aged 27 to 42	
ESR members: overall	1307	22,870	31,726	58,511	114,414
	1%	20%	28%	51%	100%
Male members	1132	16,541	20,108	34,651	72,432
	87%	72%	63%	59%	70%
Female members	175	6329	11,618	23,860	41,982
	13%	28%	37%	41%	30%

Most available data on the demographics of women in leadership in Radiology comes from the USA. According to a survey conducted by the American College of Radiology, within the aggregate of academic and private practice, 14% of men but only 7% of women are considered “leads” [9]. A recent review of 51 major US academic radiology department faculty rosters showed that women constituted 34% of academic radiologists, and the proportion of residency programme directors who were female (37%) was similar. However, the proportion of women declined at higher levels of leadership, reaching just 25% among vice chairs and section chiefs and 9% among chairs [10].

Data available from Europe suggests a similar gender gap in leadership. According to information gathered by the ESR, 20.6% of chair positions are held by women (based on research undertaken by the European Society of Radiology, 2019).

Editorial boards of scientific publications constitute another arena of leadership with a notable gender disparity. Major journal mastheads have fewer women in editorial roles than would be expected based on their representation in academic radiology [11]. Women continue to make up a minority of authors of papers in major journals, although female authorship is generally on the rise [12, 13]. An analysis of articles published in 1993, 2003 and 2013 in *Radiology*, the *American Journal of Roentgenology*, *European Radiology* and *Investigative Radiology* found that female first and senior authorship grew over the past two decades in proportion to the increasing number of practicing female radiologists [12]. Similarly, an analysis of the journal of the French Society of Radiology (*Diagnostic and Interventional Imaging*) showed that over the last three decades, the percentages of publications with female first authors and female senior authors increased from 12 to 34% and from 11 to 20%, respectively [13]. However, this upward trend has not been consistent across radiological subspecialties, and the overall proportion of female authors varies widely among subspecialties. For example, the latter has been found to be as high as 64.2% in breast imaging but just 18.5% in vascular and interventional subspecialties [14, 15].

Overall, female senior authorship has remained significantly lower than female first authorship over two decades, which likely reflects the lower representation of women in leadership positions, given the link between senior authorship and senior faculty rank [12]. Interestingly, female first authorship is associated with female last authorship, which could be an indicator of successful mentoring of junior women faculty by senior female colleagues [3, 14, 16]. This finding contrasts with the notion of a “Queen Bee syndrome”—a metaphor that has been used to describe the observation that women in positions of authority view or treat their female subordinates especially critically, thereby contributing to the underrepresentation of women in science [17]. It appears this behavioral pattern is becoming less prevalent as more women are ascending to leadership positions and generational attitudes are evolving.

It has been asked whether women advance through the ranks at the same pace as men and whether it is possible that the current gender disparity in leadership positions reflects a time lag [18]. A study published in *Radiology* found that in 2014, US academic radiologists with the same academic achievements were equally likely to be full professors, whether they were men or women [19]. These results were obtained after adjustment of variables considered reflective of academic productivity such as publications, clinical trial participation and NIH funding. Whether we should look at the adjusted or unadjusted rate of promotion to associate and full professorship remains a matter of debate [20].

## Why are women radiologists still underrepresented in leadership positions?

There is a nearly universal trend whereby female representation in Radiology decreases at increasing levels of leadership. A similar trend has also been observed in other medical specialties [21], outside medicine and in the corporate world [22]. The declining proportion of women ascending to the echelons of leadership has been compared metaphorically to a “leaky pipeline”, in which a mechanical pipeline’s fluid volume serially diminishes over its length as its contents leak out. To prevent the loss of women on career paths from medical school to academia and leadership in Radiology, it is essential to understand its causes.

### Lack of role models and mentors

The lower proportion of female leaders in medicine overall and in Radiology suggests that motivating role models and female mentors are still lacking for female medical trainees and junior faculty. As discussed more extensively later in this paper, mentorship is often a critical ingredient in the development of successful academicians and leaders.

### Generational changes: are leadership positions losing their appeal?

**Fig. 2:**
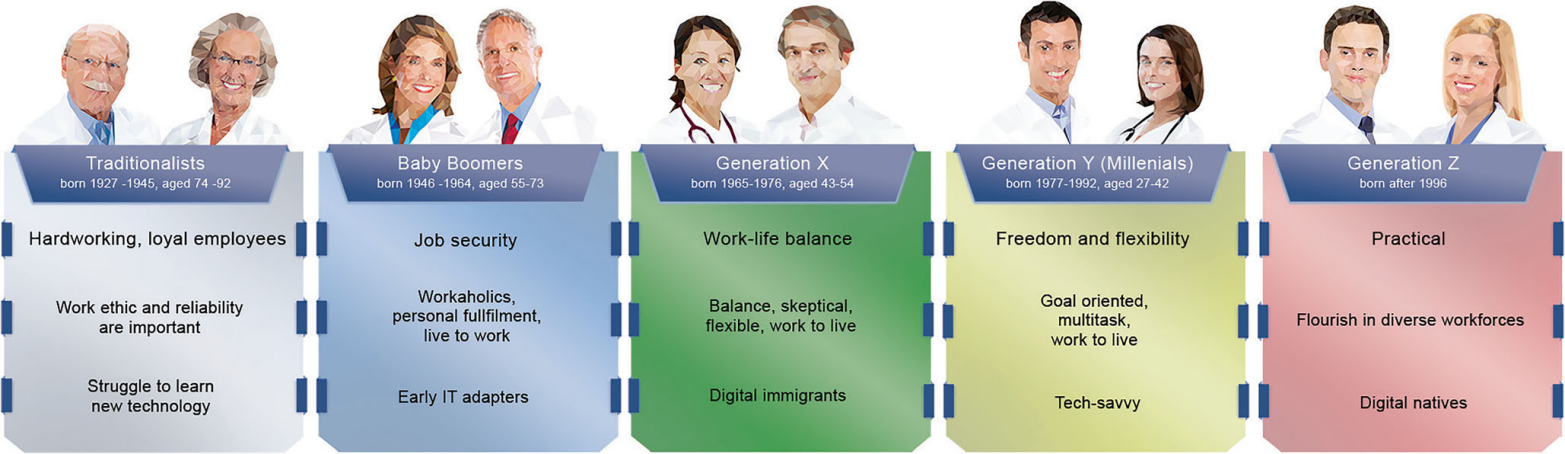
Different generations in the workplace. Future leaders will belong to generation Y or even Z. Independent of gender, the new generations have different goals and aspirations. (Based on various sources, including [23–26])

As shown in Table 1, the percentage of female ESR members is higher in the younger generations and highest in generation Y, who exhibit different goals and aspirations than older generations (Fig. 2) [23–26]. The priorities of generation Y include freedom, flexibility and work-life balance. Thus, the loss of interest in leadership may be in part generational rather than strictly gender-driven [27].

### Societal, biological and cultural obstacles for women

**Fig. 3:**
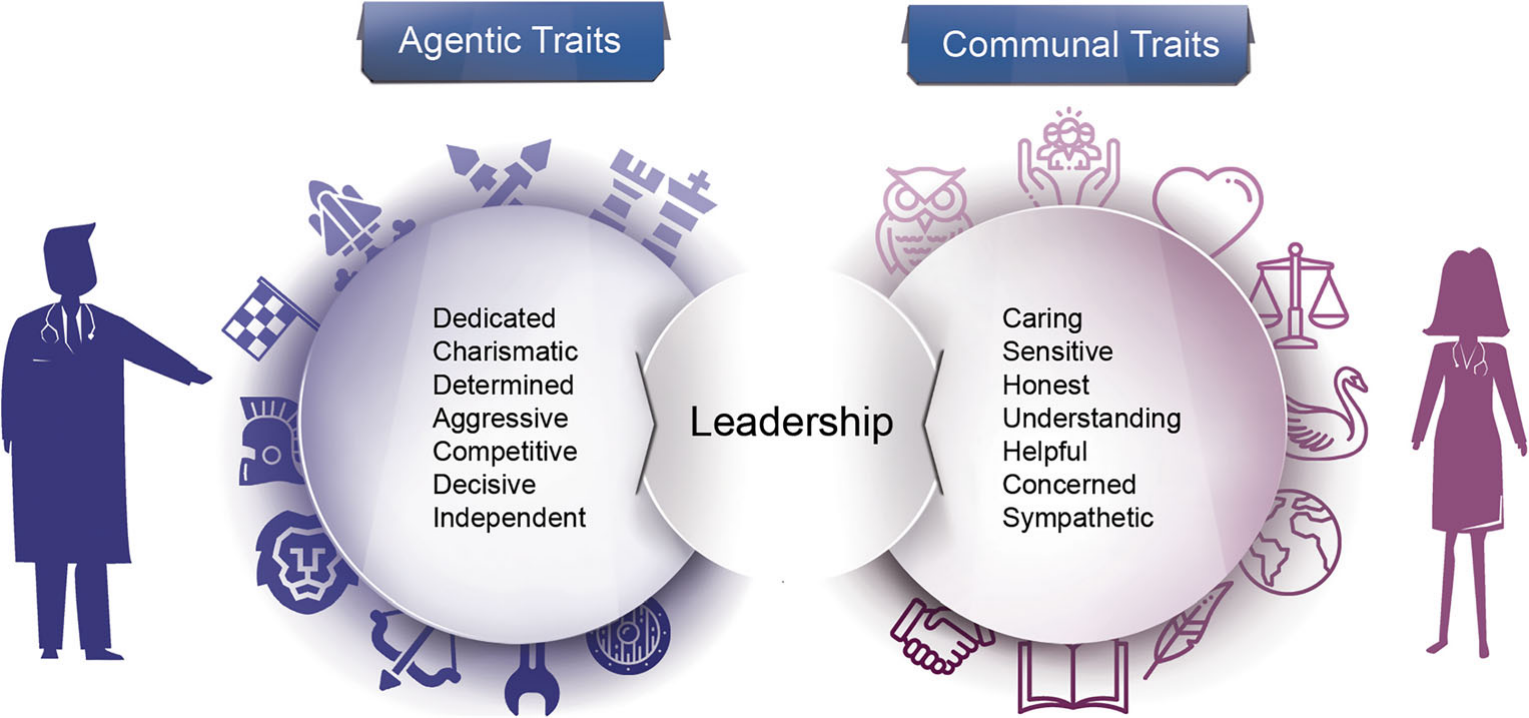
Demand-role congruity theory: agentic and communal traits associated with male and female gender, respectively (based on Eagly et al [28])

Women may encounter difficulty rising to leadership positions in the workplace when they are perceived less positively than their male counterparts. Role congruity theory, in terminology introduced by Eagly and Karau, posits that individuals are more positively evaluated when their traits align with their typical societal roles [28]. Many of the expectations about the behavior of men and women have been ascribed on a societal level and transmitted through generations. Men represented most of the workforce after the turn of the twentieth century, at a time when scientific management theory dominated industrial thinking and the concept of a corporate leadership group separated from labourers was introduced. These leaders, almost entirely men at the time, were admired societally as exhibiting *agentic* traits of decisive independence, determination, competitiveness and charisma. In contrast, women were deemed more desirable when perceived as having *communal* traits of being sensitive, caring, helpful and sympathetic (Fig. 3) [28]. Because men dominated in the leadership of the workforce, agentic personality characteristics became synonymous with the behaviors requisite for leadership success. By the same token, women who adopted agentic behaviors to match societal norms of leadership were looked upon as less female, creating a “double bind” for women in leadership that has persisted. Today, even when placed in leadership roles, women more frequently assume positions that are traditionally seen as requiring empathy, such as educational coordinators or residency programme directors [10, 29].

Women, because of their societally ascribed communal personality traits, are sometimes not believed to have the necessary skills to be competent. They may thus present themselves self-critically and reservedly, or not present themselves for leadership positions at all. Male applicants, in contrast, are often admired for their self-confidence and assertiveness and are more likely to present themselves as desirable for positions. It is therefore incumbent upon employers to become aware of their own unconscious biases as well as the unconscious reservations of potential female appointees. In particular, it is important to recognize that women leaders may not fit the traditional mould of male leaders. Female leaders need not adopt stereotypically masculine behaviors to be effective; in contradistinction, they may be more influential and inspiring leaders through emphasis of traditionally feminine traits.

Family situations may also frequently present a barrier to women’s long-term success in academic medicine. Many women with children take responsibility for a greater share of family management and work part time. Childbearing and childrearing may be seen as a burden on departmental operations and as expensive by employers and co-workers. In addition, attitudes within families may unfairly affect women. Promotion to a top job conflicts with traditional communal gender roles in the household, and some partners wrestle with the fact that their wives are more successful than themselves and that the understanding of roles in marriage may have to be renegotiated. Corroborating this idea, a widely acclaimed Swedish study examining gender differences in political leadership found that the divorce rate of women politicians was significantly higher among women who won rather than lost an election for high office [30]. Some successful women may deliberately renounce promotions they are entitled to or have been offered, bringing their personal “glass ceilings” from home to the workplace [30, 31].

It is possible that the experience or even the anticipation of conflicts in personal and familial relationships contributes to many women limiting their ambition to climb in rank [31]. Shollen et al found that 73% of men had a clear expectation for how their careers should evolve over the next 5 to 7 years and a strategy to achieve their goals, while only 50% of women had similarly well-defined plans [32].

## What strategies might foster more women leaders?

The myriad reasons leading to underrepresentation of women in leadership within academic medicine suggest that multiple long-term strategies will be necessary to solve the problem.

### Role models and mentors

Mentoring can make an important, even decisive, contribution to the personal and professional development of an academic physician. It involves a relationship between two adults in which one (the mentor) helps the other (the mentee) “to navigate the world” [33]. Mentoring provides career-related support by advising, sponsoring, exposing, coaching, protecting and encouraging an individual in his or her attempt to climb up in organizational hierarchies. It also provides psychosocial support, with the mentor helping the mentee develop competence, identity and role effectiveness by serving as a role model and offering counselling or even friendship [33–35].

**Fig. 4:**
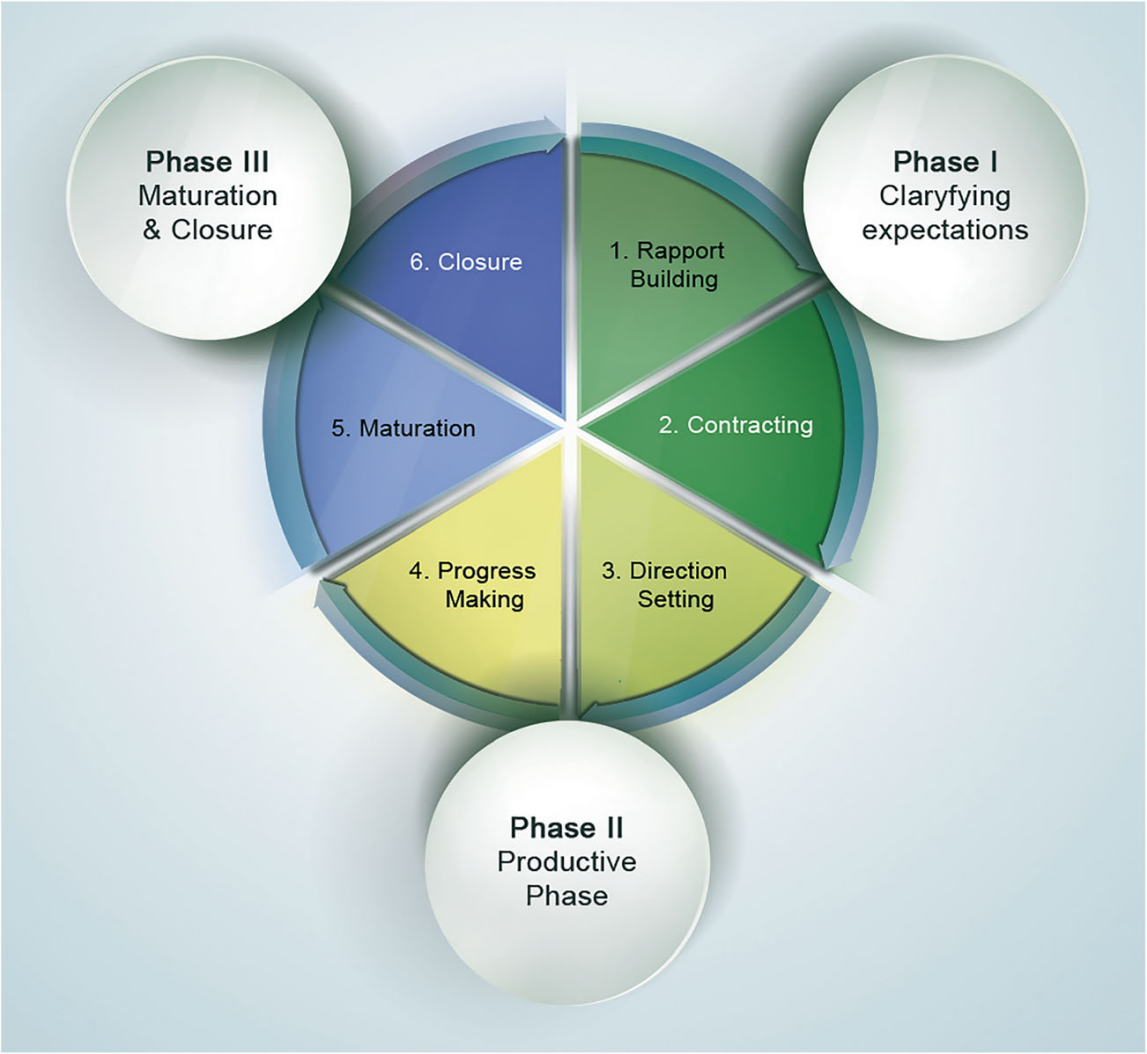
The mentoring cycle (adapted with permission from UCD People Development & Organisation Effectiveness, Human Resources, University College Dublin, http://www.ucd.ie/hr)

Mentoring can occur on a voluntary basis or through formal mentoring programmes that then may be carried out at a personal (one on one) or group level [36]. Formal mentoring programmes may even be based on a signed agreement, and a mentoring cycle consisting of distinct phases may be outlined (Fig. 4). Based on the opinion that mentoring cannot be legislated, formal mentoring programmes are not uniformly accepted but may nevertheless be helpful at the beginning of a career.

While beneficial for both men and women, mentoring may be especially important for encouraging women to ascend in their careers. Personal relationships with committed mentors can help women overcome obstacles, gain information and insights, obtain access to resources and recognize opportunities for promotion. It has been argued that mentoring is most effective when female mentees are mentored by female mentors [34]. Social identity theory, in particular, suggests that female mentors may provide more psychosocial benefits to female mentees [34, 37]. However, due to the lower number of females in higher positions, women often have mentors with “less organizational clout” [38]. Indeed, research suggests that men help women gain higher-level positions and higher salaries, and women view them as better sponsors due to their connections.

While cross-gender mentorship may be very effective and, given the shortage of female mentors, unavoidable, the relationship may be prone to resulting in gossip, envy, suspicion, speculation and even charges of sexual harassment. The negative outcomes associated with cross-gender mentoring may discourage men and women from participating in cross-gender mentorship programmes [39].

### Leadership training programmes

Leadership programmes are another effective approach to help cultivate leadership skills, independent of gender. Opportunities for women to participate in traditional leadership and management courses are sometimes limited by the restriction of admittance to candidates of specific ranks [40]. When looking to hire or promote, experience is often cited as the most important attribute of potential future leaders [5]. This narrow view can be an obstacle to increasing gender representation in leadership circles, and a more successful approach may be to look at promise when evaluating talent, particularly in the domains of curiosity, insight, determination and engagement. Targeting enrolment of women in leadership courses that cultivate talent will be essential to recruiting more female leaders.

### Unconscious bias training

Unconscious bias training seminars for those responsible for hiring and promoting have been recommended to avoid discrimination [2, 41]. Employers should commit to recruitment goals, grade applications on the same scale and blind applicants’ identities on applications.

### Gender quotas

Many parliaments have implemented some type of gender-based electoral quota. Opponents often object that use of a gender quota runs contrary to meritocracy. Proponents recommend it to quickly achieve an appropriate level of diversity. They also point out that gender disparities have not noticeably improved in the past decade and will not passively self-correct. As we have seen, the lack of women in leadership can in itself be discouraging to young women, creating a vicious cycle.

Besley et al illustrate the potential effectiveness of direct interventions to alter the gender balance in government leadership based on observations from Sweden [42]. It was assumed that mediocre male party leaders would support less qualified men in order not to be challenged by competent competitors and thus to retain their power [42]. Therefore, in 1993, the Swedish Social Democratic party implemented a “zipper quota” so that male and female candidates had to alternate on the ballot. This reform enhanced the competence of not only entire political bodies but also male politicians. Such measures might also be considered for leadership bodies in radiology.

### Recruiting

In most countries, there is a severe shortage of radiologists, and it is essential to recruit as many talented young physicians to the field as possible. Given that women generally make up half or more of medical school enrolees, this requires aggressively marketing radiology to talented female medical students [10]. “Women in radiology” groups should be formed that include female medical students interested in radiology and may be a promising means for women to build relationships, network and learn from one another.

The need for aggressive recruitment of women applies equally or even more so to the path of the clinician-scientist. This is not only because qualified young scientists are especially scarce and are essential to translate findings from basic research into clinical applications, but also because the presence of women clinician-scientists is needed to help ensure that the concerns of both genders are adequately and innovatively addressed in research. To successfully attract young women to radiology, we must work hard to make sure it is perceived as a woman- and family-friendly field that is rich in opportunities. According to Shollen et al, part-time tenure-track positions, on-site and emergency childcare and formal parental leave policies could help support the career development of women, and it thus seems likely they would aid recruitment [32].

### Networking and social media

Social media presents a new landscape for professional communication that offers numerous advantages over traditional networking, several of which can be beneficial to the elevation of women. First and perhaps most importantly, the public accessibility of thought leaders via social media lowers barriers to accessing leaders in hierarchical networks. Electronic communication makes it easier for individuals to reach out and interact with leaders and gain a reputation in their own right. Social media tools serve as highly effective platforms for dissemination, advocacy and branding with a broad reach. They have also permitted the organic development of “virtual” communities and mentors, such as the popular #RadXX handle on Twitter.

## Conclusion

It is widely understood that gender diversity enhances organizational effectiveness. However, though women account for roughly 50% of medical school enrolees, their representation in the higher ranks of academia and other realms of leadership in Radiology is much lower. A number of societal, generational and interpersonal factors underlie this disparity. A wide variety of steps can and should be taken to increase the recruitment and retention of women in Radiology. It is key to mentor and nurture female academic leaders at the early stages of their careers.

## Abbreviations

ESR: European Society of Radiology
MD: Doctor of medicine
NIH: National Institutes of Health
UCD: University College Dublin
US: United States of America
